# Characterization of Hemagglutinin Negative Botulinum Progenitor Toxins

**DOI:** 10.3390/toxins9060193

**Published:** 2017-06-15

**Authors:** Suzanne R. Kalb, Jakub Baudys, Theresa J. Smith, Leonard A. Smith, John R. Barr

**Affiliations:** 1 Centers for Disease Control and Prevention, National Center for Environmental Health, Division of Laboratory Sciences, 4770 Buford Hwy, NE, Atlanta, GA 30341, USA; skalb@cdc.gov (S.R.K.); jbaudys@cdc.gov (J.B.); 2 Molecular and Translational Sciences Division, United States Army Medical Research Institute of Infectious Diseases (USAMRIID), Ft. Detrick, MD 21702, USA; theresa.j.smith.ctr@mail.mil; 3 Office of the Chief Scientist, Medical Research and Materiel Command (MRMC), Fort Detrick, MD 21702, USA; leonard.a.smith@comcast.net

**Keywords:** botulinum neurotoxin, botulism, protein toxin, toxin complex, proteomics

## Abstract

Botulism is a disease involving intoxication with botulinum neurotoxins (BoNTs), toxic proteins produced by *Clostridium botulinum* and other clostridia. The 150 kDa neurotoxin is produced in conjunction with other proteins to form the botulinum progenitor toxin complex (PTC), alternating in size from 300 kDa to 500 kDa. These progenitor complexes can be classified into hemagglutinin positive or hemagglutinin negative, depending on the ability of some of the neurotoxin-associated proteins (NAPs) to cause hemagglutination. The hemagglutinin positive progenitor toxin complex consists of BoNT, nontoxic non-hemagglutinin (NTNH), and three hemagglutinin proteins; HA-70, HA-33, and HA-17. Hemagglutinin negative progenitor toxin complexes contain BoNT and NTNH as the minimally functional PTC (M-PTC), but not the three hemagglutinin proteins. Interestingly, the genome of hemagglutinin negative progenitor toxin complexes comprises open reading frames (orfs) which encode for three proteins, but the existence of these proteins has not yet been extensively demonstrated. In this work, we demonstrate that these three proteins exist and form part of the PTC for hemagglutinin negative complexes. Several hemagglutinin negative strains producing BoNT/A, /E, and /F were found to contain the three open reading frame proteins. Additionally, several BoNT/A-containing bivalent strains were examined, and NAPs from both genes, including the open reading frame proteins, were associated with BoNT/A. The open reading frame encoded proteins are more easily removed from the botulinum complex than the hemagglutinin proteins, but are present in several BoNT/A and /F toxin preparations. These are not easily removed from the BoNT/E complex, however, and are present even in commercially-available purified BoNT/E complex.

## 1. Introduction

Botulinum neurotoxins (BoNTs) are toxic proteins created by *Clostridium botulinum* and some related clostridia. Exposure to BoNT causes the disease known as botulism, typified by a descending flaccid paralysis which can be fatal if untreated. BoNT is comprised of two chains joined by a disulfide bond—a heavy chain which binds to receptors of neurons, and a light chain that cleaves proteins needed for nerve impulse transmission. BoNTs consist of seven confirmed serotypes, A-G, based upon antisera response. Most serotypes also display genetic and amino acid variance inside the serotype, and this variance is defined as subtype, designated by a number after the letter, as in subtype BoNT/E3. Most *C. botulinum* strains produce only one serotype of toxin; however, some strains of *C. botulinum* create more than one serotype of BoNT. These are known as bivalent toxin producers and are designated as Ab, Ba, Af, or Bf, where the capital letter designates what is believed to be the major serotype produced. 

BoNTs are produced within a protein complex called the progenitor toxin complex (PTC), made of the neurotoxin and neurotoxin-associated proteins (NAPs). The function of these NAPs is not completely understood; however, it is thought that the NAPs protect the neurotoxin from harsh conditions of the stomach and digestive tract, especially low pH and digestive enzymes [1,2,3,4]. Additionally, it is thought that these NAPs may assist with translocation of the neurotoxin through the intestinal epithelium [5,6,7,8,9], and the NAPs may aid in immunogenicity of BoNT/A [10]. The composition of the progenitor toxin can differ between serotypes, and in the case of BoNT/A, between subtypes.

The minimally functional PTC (M-PTC) of all serotypes and subtypes contains the neurotoxin and a protein of similar molecular weight known as the non-toxic non-hemagglutinin (NTNH) protein. The addition of only NTNH affords BoNT some protection from the severe conditions of the GI tract, as the oral toxicity of BoNT complexed with NTNH is increased compared to BoNT alone [11,12]. In some cases, though, there are additional proteins which comprise the PTC. BoNT/B, /C, /D, /G, and some subtypes of BoNT/A are also complexed with hemagglutinin (HA) proteins. There are three HA proteins, HA-70, HA-33, and HA-17, so named for their approximate molecular weight and the fact that HA-70 on its own is able to hemagglutinate. Strains which contain the genes for the HA proteins are termed *HA* positive (*HA*+); however, not all *C. botulinum* strains which produce BoNT are *HA*+. Strains which do not contain genes for the HA proteins do contain three open reading frames (orfs) in place of the *ha* genes, termed *orfX1*, *orfX2*, and *orfX3*. These strains are called *orfX*+. Two additional genes, *botR* and *p47*, can also be present in some strains; botR produces a protein of 21–22 kDa which binds to promoter regions to activate toxin operons, thus stimulating toxin production [13]. The function of the proteins encoded by *orfX1-3* and *p47* is unknown.

While the existence of HA-70, HA-33, and HA-17 as part of the progenitor toxin complex has been demonstrated repeatedly, there has been very little evidence for the existence of proteins encoded by *orfX1*, *orfX2*, *orfX3*, and P47. In 2005, a mass spectrometric characterization of botulinum progenitor toxins reported that the BoNT/E3 complex contains a protein encoded by *orfX1* [14]. A more recent study from 2010 reports that the PTC of BoNT/A2 contains proteins encoded by both *orfX2* and *orfX3*, but that these proteins are not present in the purified toxin complex, which consists only of BoNT/A2 and NTNH [15]. The existence of a protein encoded by *orfX2* complexed with BoNT/A2 was further verified two years later [16]. Additionally, other research findings such as the similarity between the size of the complex of BoNT/E (*orfX*+) and HA+ BoNT/A suggest the existence of proteins encoded by *orfX* genes, at least in the case of BoNT/E [17]. However, there has not been an extensive characterization of the protein composition of the progenitor toxin complexes from multiple *orfX*+ *C. botulinum* strains.

Our laboratory has successfully characterized multiple BoNTs using mass spectrometry for protein discovery, differentiation, and quantification. In 2005, we reported on our ability to differentiate two subtypes of BoNT/A, BoNT/A1 and /A2, spiked into milk [18]. Although BoNT/A1 and /A2 are about 90% identical in amino acid identity, by extracting the toxins spiked into milk, digesting the extracted toxins, and analyzing the resultant peptides by MS/MS (tandem mass spectrometry), these two similar proteins could easily be differentiated. In 2012, we reported on our ability to use mass spectral analyses to differentiate five subtypes of BoNT/B; BoNT/B1–/B5, as well as multiple toxin variants in subtypes BoNT/B1 and /B2 [19]. Additionally, we were able to use toxin proteomics for the de novo identification of a new BoNT/B subtype, BoNT/B7. Finally, in 2014, we reported on the use of mass spectrometry to detect and differentiate three different neurotoxins, BoNT/A2, /F4, and /F5, all existing in a single strain of *C. botulinum* [20]. This work also reported on the use of label free MS^E^ quantification to determine the ratio of the three toxins in a culture supernatant from *C. botulinum* strain Af84. 

Our laboratory decided to utilize our experience in proteomic analyses of botulinum neurotoxins to examine the protein composition of several hemagglutinin negative botulinum progenitor toxin complexes. In this work, we examined the protein composition of BoNT/E3, /F1, /A1, /A2, and /A3 progenitor toxin complexes, all of which are orfX+. We also studied progenitor toxin complexes from three bivalent *C. botulinum* strains; BoNT/A1(B), BoNT/A2b, and /A2f, which each contain at least one *orfX*+ toxin gene cluster in combination with either a *HA*+ toxin gene cluster (BoNT/A1(B) and /A2b) or another *orfX*+ toxin gene cluster (BoNT/A2f). We found evidence for the existence of the proteins encoded by *orfX1*, *orfX2*, and/or *orfX3* in many orfX+ progenitor toxin complexes that we studied. 

## 2. Results

**Figure 1:**
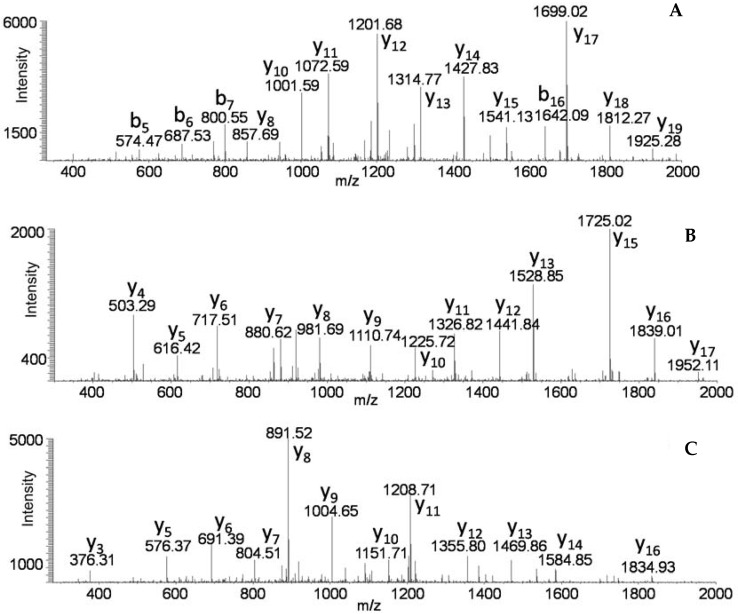
MS/MS of unique peptides TNDKDLIGTLLIEAGSSGSIIQPR from HA-70 (**A**); WLINPVSDTDETYTITNLR from HA-33 (**B**); and YLSYDNFGFISLDSLSNR from HA-17 (**C**) of BoNT/A1 (botulinum neurotoxin type A1).

The first goal was to ensure that the proposed analytical technique would capture the true nature of a botulinum progenitor toxin complex. HA*+* progenitor toxin complexes are well characterized, so the commercially-available BoNT/A1 Hall toxin complex was spiked into blank culture supernatant medium, then immunoextracted, the captured toxin complex was digested, and the resultant peptides were identified by LC-MS/MS analyses. Table 1 includes a list of all proteins identified through this process with their respective % coverages. NCBI accession numbers of the proteins identified are listed in Table S1 of the Supplementary Information. The PTC of *C. botulinum* HA+ A1 strains have been previously identified as containing BoNT/A1, NTNH, HA-70, HA-33, and HA-17 [24]. Our analyses demonstrated that all five of those proteins were present, with % coverages of 89, 90, 82, 49, and 46, respectively. Percent coverage refers to the number of amino acids from the identified peptides divided by the entire number of amino acids of the protein. Many peptides were identified which are unique to HA proteins. The MS/MS of three peptides unique to HA proteins originating from HA-70, HA-33, and HA-17 are in Figure 1.

**Figure 2:**
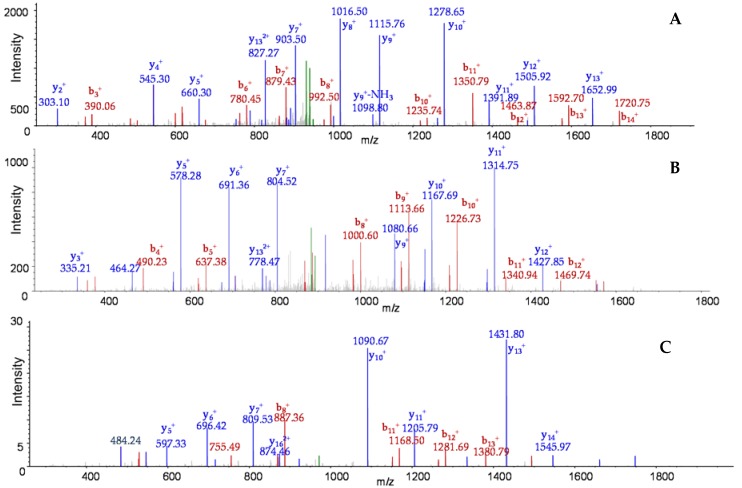
MS/MS of unique peptides NKFNLYVINEDIEKR from Orf-X1 (**A**); FTQIFSYILLNETSK from Orf-X2 (**B**); and TQSDNPEDPAIIVISSYK from Orf-X3 of BoNT/E3 (**C**). *Y*-ions are depicted in blue, *b*-ions are depicted in red, and the precursor ions are depicted in green.

The correct identification of the proteins in the progenitor toxin complex of BoNT/A1 Hall demonstrated that this analytical technique was an appropriate tool to characterize progenitor toxin complexes. The next step was to examine a commercially-available orfX*+* toxin complex. BoNT/E3 complex was spiked into blank culture supernatant medium, immunoextracted, the captured toxin complex was digested, and the resultant peptides were identified by LC-MS/MS analyses. Using this process, five proteins were identified as part of the toxin complex: BoNT/E3, NTNH from BoNT/E, Orf-X1, Orf-X2, and Orf-X3 with % sequence coverages of 88, 28, 74, 32, and 60, respectively, as listed in Table 1. The existence of these proteins is indicated through MS/MS analysis, and MS/MS of one peptide from each of the Orf-encoded proteins is in Figure 2. All three of these MS/MS analyses are from peptides which are unique to Orf-encoded proteins. Additionally, we looked at the only other commercially-available orfX*+* botulinum, the BoNT/F1 complex. There were four proteins identified as part of the complex; BoNT/F1, NTNH, Orf-X2, and P47, as listed in Table 1. 

Next, toxins from culture supernatants were immunocaptured and investigated. The digest of *C. botulinum* strain A1 *orfX*+ CDC 297 contained two proteins: BoNT/A1 and NTNH with sequence coverages of 59% and 37%, respectively, as listed in Table 1. MS/MS data was able to differentiate the /A1 orfX1+ neurotoxin from other closely related BoNT/A1 neurotoxins. Similar procedures were followed for BoNT/A2 and /A3 with comparable results. In those cases, MS/MS data identified the presence of the neurotoxin and NTNH as the constituents of the progenitor toxin complex, as seen in Table 1. Sequence coverages of 59% and 56%, respectively, were obtained for BoNT/A2; whereas coverages were lower for BoNT/A3, with 35% and 18%, respectively. Nonetheless, the MS/MS data allowed for an identification of the correct neurotoxin in these cases. The digest of *C. botulinum* strain ATCC 9564 produced similar results to that of commercially-available complex toxin, namely, that BoNT/E1, NTNH, Orf-X1, Orf-X2, and Orf-X3 were all present with sequence coverages of 91.3%, 50.5%, 85.4%, 55.1%, and 70.3%, respectively. 

**Figure 3:**
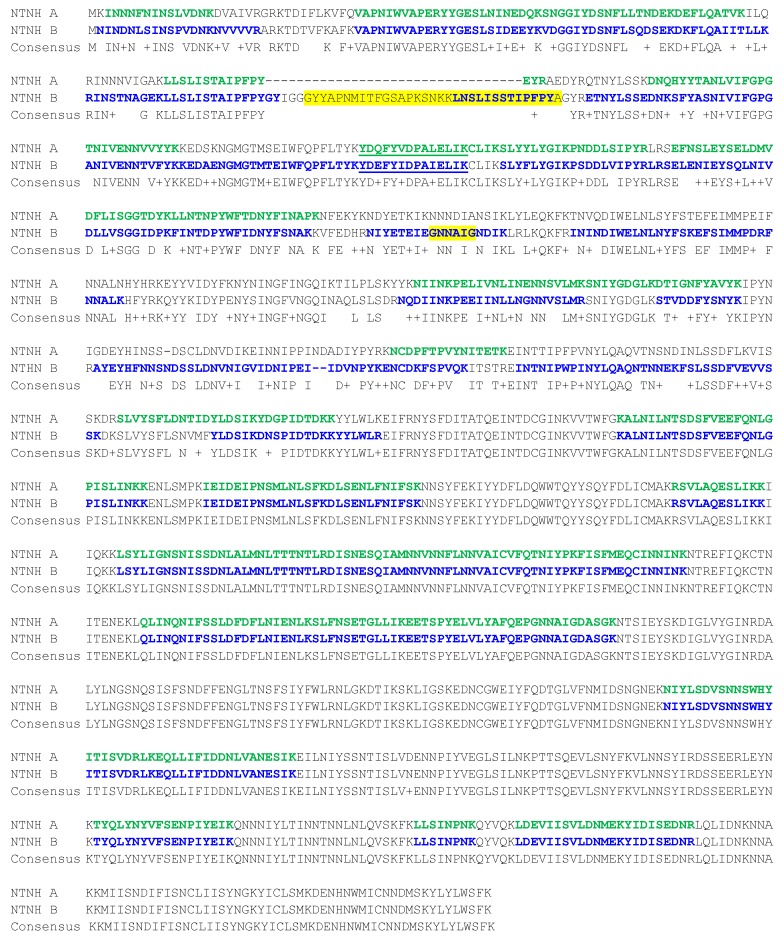
Sequence alignment of the NTNH proteins associated with BoNT/A1(B). The sequence coverage of NTNH-A is depicted in bold and green, and the sequence coverage of NTNH-B is depicted in bold and blue. The MS/MS of the underlined peptides are in Figure 4, and the sections largely responsible for binding to HA-70 in BoNT/A HA+ progenitor toxin complexes are highlighted.

**Figure 4:**
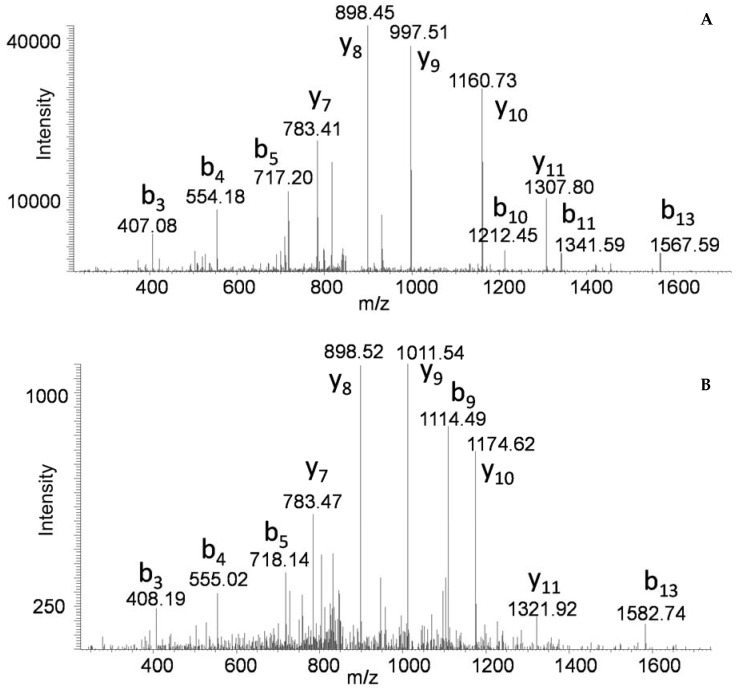
MS/MS of peptide YDQFYVDPALELIK from NTNH associated with the *bont/A1* cluster (**A**), and peptide YDEFYIDPAIELIK from NTNH associated with the *bont/(b)* cluster (**B**).

Some of the bivalent toxin producers contain mixed toxin gene clusters. An interesting example is BoNT/A1(B), as it contains a BoNT/A1 with an *orfX+* gene cluster and a BoNT/B which is not produced in its entirety due to a stop codon mutation in the gene sequence of the neurotoxin. The “silent” *bont/B* gene is part of a cluster which contains *ntnh*B and *ha* genes. Beads coated with anti-BoNT/A were added into the culture supernatant, and the extracted toxin was digested. BoNT/A1 was identified with 81% sequence coverage, along with a number of other proteins. NTNH associated with BoNT/A1 was identified (50% sequence coverage) as was NTNH associated with BoNT/B (71% sequence coverage). The sequence alignment and sequence coverage of these two proteins are seen in Figure 3. MS/MS of similar peptides YDQFYVDPALELIK from the BoNT/A NTNH (Figure 4A) and YDEFYIDPAIELIK from the BoNT/B NTNH (Figure 4B) demonstrate that both proteins are extracted with the immunocaptured BoNT/A1 and both of these peptides are similar, yet different enough for differentiation. Additionally, all three hemagglutinin proteins associated with BoNT/B were identified with the immunocaptured BoNT/A1 with specific sequence coverages: HA-70 (44%), HA-33 (28%), and HA-17 (19%), as well as Orf-X2 (14%) from the BoNT/A gene cluster.

Another bivalent toxin containing both *HA+* and *orfX+* positive genes was investigated, A2b5 CDC 1436. This bivalent toxin strain makes two active toxins. Toxin from this culture supernatant was extracted with antibodies to BoNT/A, and several proteins were identified as making up the complex: BoNT/A2 (68% sequence coverage), NTNH from BoNT/A (38%), NTNH from BoNT/B (9%), Orf-X2 (39%), Orf-X1 (17%), and Orf-X3 (10%), as listed in Table 1. Toxin from this culture supernatant was also extracted with antibodies to BoNT/B, and there were some differences in proteins making up that complex: BoNT/B5 (69% sequence coverage), NTNH from BoNT/B (49%), NTNH from BoNT/A (18%), Orf-X2 (39%), HA-33 (17%), and Orf-X1 (17%), as listed in Table 1. Finally, a bivalent toxin containing only *orfX+* genes was investigated, BoNT/A2f4. Toxin complex extracted from this culture supernatant using anti-A generated several proteins after digestion and LC-MS/MS analysis. These proteins include BoNT/A2 (74% sequence coverage), NTNH from BoNT/A (63%), NTNH from BoNT/F (50%), Orf-X2 from BoNT/A (50%), Orf-X2 from BoNT/F (51%), P47 from BoNT/A (46%), Orf-X3 from BoNT/A (20%), and Orf-X1 from BoNT/A (15%), as listed in Table 1. Other proteins were found associated with anti-BoNT/F raised against BoNT/F, which is expected to extract the BoNT/F4 toxin, as BoNT/F4 is the closest relative to BoNT/F1. These proteins include BoNT/F4 (56% sequence coverage), NTNH from BoNT/A (53%), NTNH from BoNT/F (39%), Orf-X2 from BoNT/A (51%), Orf-X2 from BoNT/F (36%), P47 from BoNT/A (48%), Orf-X3 from BoNT/A (24%), Orf-X3 from BoNT/F (18%), and Orf-X1 from BoNT/A (20%), as listed in Table 1.

Prior to this point, investigation of the hemagglutinin negative botulinum neurotoxin progenitor toxins consisted of immunoaffinity extraction of the progenitor toxin complex, followed by washes with PBST, a mild buffer. Previous work in our laboratory has established that washing the antibody-coated beads with 2 M NaCl, a stringent buffer, results in a decrease of proteins which are non-specifically bound [25]. Three hemagglutinin negative botulinum neurotoxin PTC (CDC 2357, SU1304, and ATCC 9564) were examined following washing with the stringent 2 M NaCl buffer, and the results were found to be different than progenitor toxins washes obtained previously under less stringent conditions for CDC 2357 and SU 1304. Specifically, in both cases, there is mass spectrometric evidence for the existence of the neurotoxin, the NTNH, and in the case of HA+ toxins, HA-70, HA-33, and HA-17 proteins, but there is no evidence for any of the proteins encoded by open reading frames Orf-X1, Orf-X2, or Orf-X3. All three of those proteins were present, however, in the progenitor toxin complex of ATCC 9564 even with stringent washing.

## 3. Discussion

Although the role of the classic neurotoxin-associated proteins (NAPs) is not completely understood, it is believed that these proteins assist in increasing the toxicity of botulinum neurotoxin when administered through the oral route [1]. These proteins may serve a role in shielding the neurotoxin from the severe conditions of the gastrointestinal tract and may aid in moving the neurotoxin across the intestinal epithelium through interaction with E-cadherin [2,3,7]. Accurate identification of the proteins present during each stage of intoxication would help to understand this process more fully. Additionally, because the composition of the neurotoxin-associated proteins differs between serotypes, and in some cases, subtypes, accurate identification of the neurotoxin-associated proteins can assist in further verification on the identification of the serotype/subtype of neurotoxin.

This study examined the composition of several hemagglutinin negative botulinum neurotoxin complexes, and in most cases, there was evidence for at least one of the proteins encoded by open reading frames. In the case of BoNT/E3, we observed proteins corresponding to *orfX1*, *orfX2*, and *orfX3*, all of the three open reading frames. The existence of a protein encoded by *orfX1* in this complex is supported by a 2005 report of the same finding [14]. Advances in analytical techniques and instruments might explain why the 2005 report mentioned nothing about the existence of the proteins encoded by the other open reading frames, *orfX2* and *orfX3*. Additionally, others have noted that the complex of BoNT/E although HA negative, is similar in size to HA positive complexes such as HA positive BoNT/A [17]. If the presence of a protein is not observed, this does not mean that the protein is not present. Indeed, although we did not observe *orfX* proteins in the extracted complexes in some of the strains studied here such as CDC 297, SU 1887, and Loch Maree, this does not mean that *orfX* proteins are not actually present in those complexes, but could not be detected at this point in time. Additionally, this is not the first report of the existence of a protein encoded by *orfX2*, although previous reports have found the Orf-X2 in the progenitor toxin complex [15,16], but absent in the purified toxin complex [15]. Our findings support the existence of the proteins encoded by open reading frames in hemagglutinin negative PTC and our findings from one experiment (exposure to 2 M NaCl) also support the absence of these proteins in purified toxin complex, as reported previously [15].

The creation of purified toxin complex from progenitor toxin is a rigorous process involving acid precipitation to concentrate the toxin, solubilization in sodium phosphate buffer, alkaline treatment, and precipitation with ammonium sulfate [26]. This purified toxin complex has been reported to be absent of any proteins encoded by open reading frames, at least in the case of BoNT/A2 [15], and it is likely that these proteins are removed from the toxin complex during its treatment with 3 *N* sulfuric acid and 1 *N* sodium hydroxide, in the same fashion that those proteins are removed from the toxin complex during exposure to 2 M NaCl in our experiments shown here. Of note, the wash steps are at neutral pH, and the complex is thought to dissociate at this pH [4]. However, in this study, the complex remained intact enough that the NAPs could easily be detected. This could be due to either the short time that the toxin is in the wash or due to steric constraints of having the toxin complex bound to antibody-coated beads.

Interestingly, the purification process of the toxin complex is the same for *HA+* BoNT, yet the HA proteins remain as part of the purified toxin complex. A close examination of the NTNH proteins associated with BoNT might help to explain this phenomenon. The NTNH proteins associated with HA+ BoNT are longer than the NTNH proteins associated with HA− BoNT, containing 35 additional amino acids at a site near the N-terminus. A structural model of the BoNT/A PTC shows that this 35 amino acid sequence, known as the nloop, functions as an attachment site for HA-70 [6]. This loop binds three HA-70 molecules, which are in turn bound to HA-17. One HA-17 molecule binds two HA-33 molecules, so that the entire HA-70/HA-17/HA-33 structure is attached via this one site on the NTNH. In theory, any NTNH that does not contain this nloop would be unable to bind HA70, and thus would be devoid of HA proteins. 

In fact, any NTNH that is missing this 35-amino acid sequence has been related to an orfX+ toxin cluster. What is less clearly understood is the deletion of the *ha* genes and their replacement with *orfX* genes in these toxin gene clusters. Perhaps the *orfX+* gene cluster preceded the *ha+* gene cluster chronologically, at least in the *orfX+* BoNT/A1 gene clusters. It has been hypothesized that the *ha+* BoNT/A1 clusters were the result of a recombination phenomenon within the *ntnh* genes that placed the BoNT/A1 neurotoxin gene and part of the BoNT/A1 *ntnh* gene within the BoNT/B (*ha+*) gene cluster [27]. The *orfX* genes were separated from the rest of the cluster as a result of this action. 

While the interactions leading to the structure of the HA+ toxin complex have been thoroughly investigated, the interactions of the toxin, NTNH, and the orfX proteins remain a mystery. It is obvious from our findings that these orfX protein interactions are not as secure as the HA protein interactions, and that they may consist of weaker binding, such as hydrophobic attractions only, that can be more easily disrupted by changes in pH or salt concentration. However, the constant presence of the *orfX* genes in *ha*− toxin clusters and identification of residual proteins in toxin complexes argue that they may serve some, as yet not understood, purpose. It may be possible that they serve as protectors during toxin expression or within natural environments, such as soil or water. However, the fragility of the orfX+ toxin complex makes further study challenging. 

The orfX proteins are easily removed during toxin purification or washing steps for diagnostic or detection assays. An exception to this finding is BoNT/E, which is an *orfX*+ BoNT, yet the proteins encoded by open reading frames remain as part of the purified toxin complex within the commercially-available BoNT/E complex. One potential explanation for this difference might be the isoelectric point (pI) of the M-PTC. BoNT/E has the highest isoelectric point of all the BoNT, with a pI consistently at or above six, whereas the pI of all other BoNT are below six. Additionally, the NTNH associated with BoNT/E also have the highest pI, with a pI consistently above five, whereas the pI of the other NTNHs are all below five. Perhaps the differences in pI could account for increased stability of the bond between BoNT/E and the proteins encoded by open reading frames.

Another interesting observation of this work is that the immunoaffinity purification of the neurotoxin from a bivalent strain appeared to purify the NTNH associated with both gene clusters. It is important to note that the antibodies used for immunoaffinity purification are directed toward the neurotoxin itself, and that in all cases examined, there were no instances of both neurotoxins purified with the antibody designed for only one serotype. The presence of both NTNHs might be explained through an understanding of the interaction between the neurotoxin and NTNH. The interactions between BoNT/A1 Hall and its NTNH have been published and consist of 25 sites throughout the sequence of NTNH, but primarily in the C-terminal half of NTNH, with 21 of the 25 sites in the last third of the neurotoxin [4]. Table 2 consists of a list of these sites in the NTNH associated with BoNT/A1 Hall. Although the interactions between BoNT/A1(B) and its NTNH have not been published, the interactions are likely similar due to the high sequence similarity of BoNT/A1 Hall and BoNT/A1(B); in fact, these sequences are 99.8% identical, with only two residues that differ between the two proteins: P1Q and A26V. Of the 21 sites of BoNT/A1 Hall known to interact with its NTNH, 100% of those sites are identical to the sequence of BoNT/A1(B). 

Looking at the sites of the NTNH associated with BoNT/A1 Hall (NTNH-A) that are known to contact BoNT/A1 Hall listed in Table 2 and the sequence homology between the NTNH-A associated with the A1 cluster of BoNT/A1(B), it is apparent that there is a high degree of similarity; 19 of the 25 sites are 100% identical, and the remaining six sites are conserved mutations, yielding 76% identity and 100% similarity. Therefore, the interactions of NTNH-A associated with BoNT/A1(B) are likely nearly identical to the interactions published between BoNT/A1 Hall and its NTNH-A. Next, a comparison of the 25 sites in the NTNH-B presumed to interact with BoNT/A1(B) shows that all 25 of these sites are 100% identical in the amino acid sequences of the NTNHs associated with both the A and B clusters within BoNT/A1(B). Therefore, because the residues of NTNH-A presumed to interact with BoNT/A1 from the A1(B) cluster are 100% identical with those of NTNH-B, the neurotoxin apparently associates with each interchangeably, yielding our discovery here of the association of the neurotoxin with the NTNH from both gene clusters. Unfortunately, the interactions of the remaining toxins used in this study, BoNT/A2, /A3, /B5, and /F4, and their NTNH have not been published, and because there is a lower degree of amino acid similarity between these neurotoxins and BoNT/A1 Hall (39–90%), it is not possible to theorize about the interaction of these neurotoxins and their NTNHs.

In conclusion, we have examined the composition of the PTC of several hemagglutinin negative botulinum neurotoxin complexes and have found that most of the *orfX*+ PTC studied here contain at least some of the proteins encoded by these open reading frames. These proteins are more easily removed from the complex than the hemagglutinin proteins because these proteins were not observed following washing of immunoaffinity purified BoNT complexes in 2 M NaCl, with the exception of the toxin complex of BoNT/E. Commercially-available purified BoNT/E complex contains all three proteins encoded by open reading frames despite the harsh conditions used to purify the toxin complex. Additionally, several BoNT/A containing bivalent strains were examined, and the NTNHs and NAPs from both gene clusters were observed following immunoaffinity purification of each toxin component.

## 4. Materials and Methods

### 4.1. Materials

CR2 and RAZ1, monoclonal antibodies to BoNT/A, BoNT/B (2B18.2 and B12.1), and BoNT/E (4E17.1) were attained from Dr. James Marks of the University of California at San Francisco [21,22,23]. Polyclonal antibodies to BoNT/F1 were procured from Metabiologics (Madison, WI, USA). Dynabeads^®^ (M-280/Streptavidin) were obtained from Invitrogen (Carlsbad, CA, USA). BoNT/A, /E, and /F complex toxins were purchased from Metabiologics (Madison, WI, USA). All chemicals were purchased from Sigma-Aldrich (St. Louis, MO, USA) unless otherwise indicated. Sulfo-NHS-Biotin was purchased from Thermo Fisher Scientific (Waltham, MA, USA). Kingfisher plates and tip combs were obtained from Thermo Fisher Scientific (Waltham, MA, USA). Trypsin (gold, mass spectrometry grade) and sequencing grade chymotrypsin were purchased from Promega (Madison, WI, USA).

### 4.2. Preparation of Ab-Coated Beads 

The Abs (20 μg) were biotinylated with 4 μL of the 300 μM sulfo-NHS-biotin in water which was prepared immediately prior to use. The Ab/biotin was incubated at room temperature overnight with no mixing. Biotin labeled Ab (2 μg total for each serotype) was added to washed streptavidin-coated beads (twice with 1 mL of Phosphate Buffered Saline and 0.05% Tween-20 (PBST); reconstituted in 100 μL PBST) and mixed, keeping beads in solution, at room temperature for 1 h. Finally, the beads were then washed two times in 1 mL each of PBST (neutral pH) and next the Ab bound beads were resuspended in 100 μL PBST. For a greater number of samples, the volumes were increased proportionally.

### 4.3. Production and Immunocapture of C. botulinum Culture Supernatants and BoNT Complexes

Biosafety Level-2 processes, practices, and facilities were used to ensure safe conditions while working with BoNT. Additionally, toxin stock material and samples containing BoNT were handled in a Class II biosafety cabinet with HEPA filters to minimize the risk of aerosol exposure. Crude culture supernatants representing *C. botulinum* strains A1 *orfX*+ CDC 297, A1(B) CDC 2357, A2f4 SU1304, A3 Loch Maree, A2b5 CDC 1436, A2 SU1887, and ATCC 9564 (additional information is available in Table 1) were produced by growing a subculture for five days at 35 °C in Tryptone Peptone Glucose Yeast Extract (TPGY) medium. After centrifugation, the supernatants were separated and filtered through 0.22 µm filters. An aliquot of the culture supernatant from 100 μL to 500 μL was positioned into a deep well plate containing PBST (either 1X or 10X) to constitute a final volume from 500 μL to 550 μL with an ultimate concentration of 1X PBST. 

To extract BoNT/A, 20 μL of beads coated with 0.4 μg of RAZ1 and CR2 mAbs were added into the culture supernatant. The deep well plate was covered and positioned on a plate shaker at the lowest speed needed to keep the beads soluble for 1 h. The deep well plate was uncapped and placed in a KingFisher Flex magnetic particle processor (Thermo Fisher Scientific, Waltham, MA, USA) used for automated bead washing, including two washes of 1 mL each of 2 M NaCl (stringent wash) or 1X PBST (gentle wash) followed by two washes of 1 mL each of 1X PBST. The beads were eluted from the KingFisher Flex in 80 μL of water and next were removed from the KingFisher Flex. Extraction of BoNT/B /E, or /F proceeded in the same fashion, using specific mAbs, as indicated in the materials section, to capture the minor component of the strains in Table 1. Negative controls consisted of culture supernatants extracted with beads conjugated with an irrelevant antibody (i.e., BoNT/B for cultures absent of BoNT/B or ricin for cultures containing BoNT/B). Positive controls were culture supernatant medium spiked with 1 μg of BoNT/A, /B, /E, or /F complexes, and diluted in PBST to necessary levels immediately before spiking, with the rest of the extraction protocol as above.

### 4.4. Digestion and LC-MS/MS Analysis of BoNTs

The immunocaptured progenitor toxin complexes were first digested with trypsin and then chymotrypsin with the same protocol previously used [20]. Essentially, the toxin complexes were digested separately with trypsin and chymotrypsin for 5 min at 52 °C. Toxin complex peptides were identified with LC-MS/MS and database searching as described previously [20], with the peptides separated on a Waters NanoAcquity C18 1.7 μm UPLC column and then analyzed in MS and MS/MS modes on a LTQ-Orbitrap Elite instrument.

## Figures and Tables

**Table 1:** Identity and sequence coverage of proteins from this study. PBST, Phosphate Buffered Saline and 0.05% Tween-20, NTNH, nontoxic non-hemagglutinin.

Strain	Serotype/Subtype	Complex/Cluster Type	Ab Used for Extraction	Source	Wash	Protein Identified	% Sequence Coverage
Hall A	A1	HA+	CR2/RAZ1 (anti-A)	Spiked	PBST	BoNT/A1 Hall	88.5
Hall A	A1	HA+	CR2/RAZ1 (anti-A)	Spiked	PBST	NTNH from BoNT/A	89.7
Hall A	A1	HA+	CR2/RAZ1 (anti-A)	Spiked	PBST	HA-70 from BoNT/A	81.6
Hall A	A1	HA+	CR2/RAZ1 (anti-A)	Spiked	PBST	HA-33 from BoNT/A	59.1
Hall A	A1	HA+	CR2/RAZ1 (anti-A)	Spiked	PBST	HA-17 from BoNT/A	46.2
Alaska E	E3	Orf+	4E17.1 (anti-E)	Spiked	PBST	BoNT/E3	88.2
Alaska E	E3	Orf+	4E17.1 (anti-E)	Spiked	PBST	NTNH from BoNT/E	28.3
Alaska E	E3	Orf+	4E17.1 (anti-E)	Spiked	PBST	Orf-X1	74.3
Alaska E	E3	Orf+	4E17.1 (anti-E)	Spiked	PBST	Orf-X2	32.1
Alaska E	E3	Orf+	4E17.1 (anti-E)	Spiked	PBST	Orf-X3	59.7
Langland F	F1	Orf+	Polyclonal anti-F	Spiked	PBST	BoNT/F1	84.1
Langland F	F1	Orf+	Polyclonal anti-F	Spiked	PBST	NTNH from BoNT/F	81.7
Langland F	F1	Orf+	Polyclonal anti-F	Spiked	PBST	P47	25.0
Langland F	F1	Orf+	Polyclonal anti-F	Spiked	PBST	Orf-X2	14.9
CDC 297	A1	Orf+	CR2/RAZ1 (anti-A)	Untreated supernatant	PBST	BoNT/A1	58.8
CDC 297	A1	Orf+	CR2/RAZ1 (anti-A)	Untreated supernatant	PBST	NTNH from BoNT/A1	36.5
SU1887	A2	Orf+	CR2/RAZ1 (anti-A)	Untreated supernatant	PBST	BoNT/A2	59.4
SU1887	A2	Orf+	CR2/RAZ1 (anti-A)	Untreated supernatant	PBST	NTNH from BoNT/A2	56.4
Loch Maree	A3	Orf+	CR2/RAZ1 (anti-A)	Untreated supernatant	PBST	BoNT/A3	34.9
Loch Maree	A3	Orf+	CR2/RAZ1 (anti-A)	Untreated supernatant	PBST	NTNH from BoNT/A3	18.0
CDC 2357	A1(B)	HA+ and Orf+	CR2/RAZ1 (anti-A)	Untreated supernatant	PBST	BoNT/A1(B)	81.2
CDC 2357	A1(B)	HA+ and Orf+	CR2/RAZ1 (anti-A)	Untreated supernatant	PBST	NTNH from BoNT/B	70.5
CDC 2357	A1(B)	HA+ and Orf+	CR2/RAZ1 (anti-A)	Untreated supernatant	PBST	NTNH from BoNT/A	50.1
CDC 2357	A1(B)	HA+ and Orf+	CR2/RAZ1 (anti-A)	Untreated supernatant	PBST	HA-70	44.4
CDC 2357	A1(B)	HA+ and Orf+	CR2/RAZ1 (anti-A)	Untreated supernatant	PBST	HA-33	28.3
CDC 2357	A1(B)	HA+ and Orf+	CR2/RAZ1 (anti-A)	Untreated supernatant	PBST	HA-17	19.3
CDC 2357	A1(B)	HA+ and Orf+	CR2/RAZ1 (anti-A)	Untreated supernatant	PBST	Orf-X2	14.4
CDC 2357	A1(B)	HA+ and Orf+	CR2/RAZ1 (anti-A)	Untreated supernatant	NaCl	BoNT/A1(B)	74.9
CDC 2357	A1(B)	HA+ and Orf+	CR2/RAZ1 (anti-A)	Untreated supernatant	NaCl	NTNH from BoNT/B	64.0
CDC 2357	A1(B)	HA+ and Orf+	CR2/RAZ1 (anti-A)	Untreated supernatant	NaCl	NTNH from BoNT/A	47.6
CDC 2357	A1(B)	HA+ and Orf+	CR2/RAZ1 (anti-A)	Untreated supernatant	NaCl	HA-70	54.2
CDC 2357	A1(B)	HA+ and Orf+	CR2/RAZ1 (anti-A)	Untreated supernatant	NaCl	HA-33	44.1
CDC 2357	A1(B)	HA+ and Orf+	CR2/RAZ1 (anti-A)	Untreated supernatant	NaCl	HA-17	38.4
CDC 1436	A2b5	HA+ and Orf+	CR2/RAZ1 (anti-A)	Untreated supernatant	PBST	BoNT/A2	68.4
CDC 1436	A2b5	HA+ and Orf+	CR2/RAZ1 (anti-A)	Untreated supernatant	PBST	NTNH from BoNT/A2	37.7
CDC 1436	A2b5	HA+ and Orf+	CR2/RAZ1 (anti-A)	Untreated supernatant	PBST	NTNH from BoNT/B	8.7
CDC 1436	A2b5	HA+ and Orf+	CR2/RAZ1 (anti-A)	Untreated supernatant	PBST	Orf-X2	38.9
CDC 1436	A2b5	HA+ and Orf+	CR2/RAZ1 (anti-A)	Untreated supernatant	PBST	Orf-X1	16.9
CDC 1436	A2b5	HA+ and Orf+	CR2/RAZ1 (anti-A)	Untreated supernatant	PBST	Orf-X3	9.6
CDC 1436	A2b5	HA+ and Orf+	2B18.2/B12.1 (anti-B)	Untreated supernatant	PBST	BoNT/B5	69.3
CDC 1436	A2b5	HA+ and Orf+	2B18.2/B12.1 (anti-B)	Untreated supernatant	PBST	NTNH from BoNT/B	48.6
CDC 1436	A2b5	HA+ and Orf+	2B18.2/B12.1 (anti-B)	Untreated supernatant	PBST	Orf-X2	39.1
CDC 1436	A2b5	HA+ and Orf+	2B18.2/B12.1 (anti-B)	Untreated supernatant	PBST	HA-33	16.7
CDC 1436	A2b5	HA+ and Orf+	2B18.2/B12.1 (anti-B)	Untreated supernatant	PBST	Orf-X1	16.9
SU1304	A2f4	Orf+	CR2/RAZ1 (anti-A)	Untreated supernatant	PBST	BoNT/A2	73.6
SU1304	A2f4	Orf+	CR2/RAZ1 (anti-A)	Untreated supernatant	PBST	NTNH from BoNT/A2	63.2
SU1304	A2f4	Orf+	CR2/RAZ1 (anti-A)	Untreated supernatant	PBST	NTNH from BoNT/F	49.8
SU1304	A2f4	Orf+	CR2/RAZ1 (anti-A)	Untreated supernatant	PBST	Orf-X2 from BoNT/A	50.0
SU1304	A2f4	Orf+	CR2/RAZ1 (anti-A)	Untreated supernatant	PBST	Orf-X2 from BoNT/F	50.5
SU1304	A2f4	Orf+	CR2/RAZ1 (anti-A)	Untreated supernatant	PBST	P47 from BoNT/A	45.9
SU1304	A2f4	Orf+	CR2/RAZ1 (anti-A)	Untreated supernatant	PBST	Orf-X3 from BoNT/A	19.8
SU1304	A2f4	Orf+	CR2/RAZ1 (anti-A)	Untreated supernatant	PBST	Orf-X1 from BoNT/A	14.8
SU1304	A2f4	Orf+	CR2/RAZ1 (anti-A)	Untreated supernatant	NaCl	BoNT/A2	75.1
SU1304	A2f4	Orf+	CR2/RAZ1 (anti-A)	Untreated supernatant	NaCl	NTNH from BoNT/A2	61.0
SU1304	A2f4	Orf+	CR2/RAZ1 (anti-A)	Untreated supernatant	NaCl	NTNH from BoNT/F	54.8
SU1304	A2f4	Orf+	Polyclonal anti-F	Untreated supernatant	PBST	BoNT/F4	56.2
SU1304	A2f4	Orf+	Polyclonal anti-F	Untreated supernatant	PBST	NTNH from BoNT/A	52.9
SU1304	A2f4	Orf+	Polyclonal anti-F	Untreated supernatant	PBST	NTNH from BoNT/F	39.3
SU1304	A2f4	Orf+	Polyclonal anti-F	Untreated supernatant	PBST	Orf-X2 from BoNT/A	50.5
SU1304	A2f4	Orf+	Polyclonal anti-F	Untreated supernatant	PBST	Orf-X2 from BoNT/F	35.7
SU1304	A2f4	Orf+	Polyclonal anti-F	Untreated supernatant	PBST	P47 from BoNT/A	48.3
SU1304	A2f4	Orf+	Polyclonal anti-F	Untreated supernatant	PBST	Orf-X3 from BoNT/A	23.7
SU1304	A2f4	Orf+	Polyclonal anti-F	Untreated supernatant	PBST	Orf-X3 from BoNT/F	17.9
SU1304	A2f4	Orf+	Polyclonal anti-F	Untreated supernatant	PBST	Orf-X1 from BoNT/A	20.4
ATCC 9564	E1	Orf+	4E17.1 (anti-E)	Untreated supernatant	PBST	BoNT/E1	91.3
ATCC 9564	E1	Orf+	4E17.1 (anti-E)	Untreated supernatant	PBST	NTNH from BoNT/E	50.5
ATCC 9564	E1	Orf+	4E17.1 (anti-E)	Untreated supernatant	PBST	Orf-X1	85.4
ATCC 9564	E1	Orf+	4E17.1 (anti-E)	Untreated supernatant	PBST	Orf-X2	55.1
ATCC 9564	E1	Orf+	4E17.1 (anti-E)	Untreated supernatant	PBST	Orf-X3	70.3
ATCC 9564	E1	Orf+	4E17.1 (anti-E)	Untreated supernatant	NaCl	BoNT/E1	86.2
ATCC 9564	E1	Orf+	4E17.1 (anti-E)	Untreated supernatant	NaCl	NTNH from BoNT/E	29.9
ATCC 9564	E1	Orf+	4E17.1 (anti-E)	Untreated supernatant	NaCl	Orf-X1	86.1
ATCC 9564	E1	Orf+	4E17.1 (anti-E)	Untreated supernatant	NaCl	Orf-X2	44.1
ATCC 9564	E1	Orf+	4E17.1 (anti-E)	Untreated supernatant	NaCl	Orf-X3	66.9

**Table 2:** Conservation of residues from NTNH (nontoxic non-hemagglutinin) reported to interact with BoNT.

NTNH from A1 Hall	Identity in A Cluster of A1(B)	Identity in B Cluster of A1(B)
K97	Identical	Identical
E342	Identical	Identical
D455	Identical	Identical
V457	Identical	Identical
C583	Identical	Identical
N652	Identical	Identical
E758	Q758 (conserved)	Q758 (conserved)
N800	Identical	Identical
Q801	Identical	Identical
V803	I803 (conserved)	I803 (conserved)
L807	Identical	Identical
D808	Identical	Identical
E810	D810 (conserved)	D810 (conserved)
F811	Identical	Identical
I814	Identical	Identical
Q815	E815 (conserved)	E815 (conserved)
E831	Identical	Identical
K844	Q844 (conserved)	Q844 (conserved)
E845	Identical	Identical
N1039	Identical	Identical
N1100	D1100 (conserved)	D1100 (conserved)
N1102	Identical	Identical
Q1107	Identical	Identical
D1110	Identical	Identical
E1111	Identical	Identical

## Supplementary Materials

The following are available online at www.mdpi.com/2072-6651/9/6/193/s1, Table S1: List of NCBI accession numbers for proteins identified.
