# Supplementary material for: Characterization of Hemagglutinin Negative Botulinum Progenitor Toxins
Source: Toxins (Basel). 2017 Jun 15;9(6):193. doi: 10.3390/toxins9060193 (PMC5488043; doi:10.3390/toxins9060193)
Supplement: Supplementary file 1 [file toxins-09-00193-s001.pdf]

Supplementary Materials: Characterization of Hemagglutinin Negative Botulinum Progenitor Toxins

Suzanne R. Kalb, Jakub Baudys, Theresa J. Smith, Leonard A. Smith, John R. Barr

Table S1. List of NCBI accession numbers for proteins identified.

Strain/Complex of Origin	Protein	NCBI Accession Number
BoNT A1 Hall	BoNT/A1 Hall	WP_011948511.1
BoNT A1 Hall	NTNH from BoNT/A	CAA63550.1
BoNT A1 Hall	HA-70 from BoNT/A	WP_011948507
BoNT A1 Hall	HA-33 from BoNT/A	WP_011948508.1
BoNT A1 Hall	HA-17 from BoNT/A	WP_003356711.1
BoNT E3	BoNT/E3	WP_012451426.1
BoNT E3	NTNH from BoNT/E3	P46082.1
BoNT E3	Orf-X1 from BoNT/E3	ALT05363.1
BoNT E3	Orf-X2 from BoNT/E3	ALT05471.1
BoNT E3	Orf-X3 from BoNT/E3	ALT05470.1
BoNT F1	BoNT/F1	WP_011987710.1
BoNT F1	NTNH from BoNT/F	WP_011987709.1
BoNT F1	P47 from BoNT/F	WP_011987708.1
BoNT F1	Orf-X2 from BoNT/F	WP_012703709.1
BoNT A1 OrfX+	BoNT/A1	ABM73969.1
BoNT A1 OrfX+	NTNH from BoNT/A1	CAA61123.1
BoNT A2	BoNT/A2	WP_012703873.1
BoNT A2	NTNH from BoNTA2	CAA61233.1
BoNT A3	BoNT/A3	WP_012301031.1
BoNT A3	NTNH from BoNT/A3	WP_012300955.1
BoNT A1(B)	BoNT/A1(B)	WP_003356619.1
BoNT A1(B)	NTNH from BoNT/B	WP_003357111.1
BoNT A1(B)	NTNH from BoNT/A	WP_003355794.1
BoNT A1(B)	HA-70 from BoNT/B	WP_003356638.1
BoNT A1(B)	HA-33 from BoNT/B	WP_003356066.1
BoNT A1(B)	HA-17 from BoNT/B	WP_012291566.1
BoNT A1(B)	Orf-X2 from BoNT/A	WP_003356338.1
BoNT A2b5 (extracted with anti-A)	BoNT/A2	WP_040110135.1
BoNT A2b5 (extracted with anti-A)	NTNH from BoNT/A2	WP_040110134.1
BoNT A2b5 (extracted with anti-A)	NTNH from BoNT/B	WP_039698966.1
BoNT A2b5 (extracted with anti-A)	Orf-X2 from BoNT/A	WP_040110130.1
BoNT A2b5 (extracted with anti-A)	Orf-X1 from BoNT/A	WP_012704051.1
BoNT A2b5 (extracted with anti-A)	Orf-X3 from BoNT/A	WP_040110129.1
BoNT A2b5 (extracted with anti-B)	BoNT/B5	WP_040110073.1
BoNT A2b5 (extracted with anti-B)	NTNH from BoNT/B	WP_012431075.1
BoNT A2b5 (extracted with anti-B)	Orf-X2 from BoNT/A	WP_040110130.1
BoNT A2b5 (extracted with anti-B)	HA-33 from BoNT/B	WP_012291520.1
BoNT A2b5 (extracted with anti-B)	Orf-X1 from BoNT/A	WP_012704051.1
BoNT A2f4 (extracted with anti-A)	BoNT/A2	WP_012703873.1
BoNT A2f4 (extracted with anti-A)	NTNH from BoNT/A2	WP_025775292.1
BoNT A2f4 (extracted with anti-A)	NTNH from BoNT/F	WP_025775292.1
BoNT A2f4 (extracted with anti-A)	Orf-X2 from BoNT/A	WP_021107445.1

BoNT A2f4 (extracted with anti-A)	Orf-X2 from BoNT/F	WP_021106991.1
BoNT A2f4 (extracted with anti-A)	P47 from BoNT/A	WP_012704373.1
BoNT A2f4 (extracted with anti-A)	Orf-X3 from BoNT/A	WP_003357203.1
BoNT A2f4 (extracted with anti-A)	Orf-X1 from BoNT/A	WP_012300974.1
BoNT A2f4 (extracted with anti-F)	BoNT/F4	WP_021106996.1
BoNT A2f4 (extracted with anti-F)	NTNH from BoNT/A	ADA79542.1
BoNT A2f4 (extracted with anti-F)	NTNH from BoNT/F	WP_025775292.1
BoNT A2f4 (extracted with anti-F)	Orf-X2 from BoNT/A	WP_012301067.1
BoNT A2f4 (extracted with anti-F)	Orf-X2 from BoNT/F	WP_021106991.1
BoNT A2f4 (extracted with anti-F)	P47 from BoNT/A	WP_012704373.1
BoNT A2f4 (extracted with anti-F)	Orf-X3 from BoNT/A	WP_012705769.1
BoNT A2f4 (extracted with anti-F)	Orf-X3 from BoNT/F	WP_076174538.1
BoNT A2f4 (extracted with anti-F)	Orf-X1 from BoNT/A	WP_040110131.1
